# Professor Joseph Leidy

**Published:** 1891-06

**Authors:** 


### PROFESSOR JOSEPH LEIDY.

THIS distinguished scientist passed from active life April 30, 1891.

He had not been in good health for some time, yet he continued his work up to within a few days before his death. He had completed the examinations at the Departments of Medicine and Dentistry of the University only a few days before his final sickness. He was not able to sign the diplomas, thus literally dying in full harness.

He had stood for many years as the leading scientist in this country. His attainments were as varied as they were profound, and it came to be understood that the last resort in the solving of difficult problems was Professor Leidy.

He was born in Philadelphia sixty-eight years ago, and very early entered into the study of mineralogy and botany. He graduated in medicine at the University of Pennsylvania in 1844. He subsequently assisted Professor Hare in his chemical laboratory. For a time during the war he served as surgeon at the Satterlee General Hospital in Philadelphia. In 1846 he was made demonstrator of anatomy of Franklin Medical College, and later made a tour of Europe. In 1853 he was appointed to the chair of Anatomy in the University of Pennsylvania, and in 1871 was elected to the chair of Natural History in Swarthmore College.

The amount of work done by Professor Leidy is too extensive to be even mentioned here. His contributions were constant as his work was untiring. He was recognized all over the world, and made a member of the leading scientific societies in almost all countries. His work for the United States government was extensive: that on the "Fresh Water Rhizopods of North America" will carry his name in scientific history as one of his most remarkable productions.

He was a fine draughtsman, and was thus enabled to illustrate his work and his lectures in the most satisfactory manner.

Those who knew Dr. Leidy intimately appreciated most fully his kindly nature, ever eager to impart information, but at the same time freer of egoism than any man the writer ever knew.

He was Professor of Anatomy in the Department of Dentistry, University of Pennsylvania, from the date of its organization. The many students, the world over, who have benefited by his thorough teaching will mourn his loss not only as a teacher but as the ever-kindly adviser.

Professor Leidy was president of the "Anthropometric Society," the main object of which is the minute examination of the brain. It is singular that Dr. Leidy and his brother, who died the day previous, furnished the material for the first examination.

They were both cremated Tuesday, May 5, 1891.

